# Draft Genome Sequence of *Listeria monocytogenes* Strain ATCC 7644

**DOI:** 10.1128/genomeA.00970-17

**Published:** 2017-09-21

**Authors:** Marc-Olivier Duceppe, Catherine Carrillo, Hongsheng Huang

**Affiliations:** a Canadian Food Inspection Agency, Ottawa Laboratory Fallowfield, Ontario, Canada; b Canadian Food Inspection Agency, Ottawa Laboratory Carling, Ontario, Canada

## Abstract

*Listeria monocytogenes* is a Gram-positive, rod-shaped, non-spore-forming bacterium which is an important foodborne bacterial pathogen for humans worldwide with high mortality rates. Here, we report a 2,964,284-bp draft genome sequence of *Listeria monocytogenes* strain ATCC 7644 (American Type Culture Collection).

## GENOME ANNOUNCEMENT

*Listeria monocytogenes* is a Gram-positive, rod-shaped, non-spore-forming bacterium and an important foodborne bacterial pathogen for humans worldwide with high mortality rates (1). *Listeria* species have been widely used in numerous research studies for biology, pathogenesis, and diagnostics (2). In particular, *L. monocytogenes* strain ATCC 7644 has been used frequently as a model organism (3, 4), but its whole-genome sequence is not available, which limits the understanding of its molecular pathogenesis. Therefore, this article reports its draft genome sequence.

*L. monocytogenes* strain ATCC 7644 (serogroup 1/2c) isolated from a human was obtained from ATCC (ATCC, Manassas, VA, USA) and stored at −80°C. Genomic DNA was isolated from overnight cultures grown at 37°C on brain and heart infusion agar using the Qiagen DNeasy blood and tissue DNA purification kit (product no. 69504, Qiagen, Toronto, Ontario, Canada). Three sequencing libraries were constructed from three independent cultures/DNA extractions using the Nextera XT DNA sample preparation kit (Illumina, Inc., San Diego, CA), and library quality was analyzed using a BioAnalyzer (Agilent Technologies, Santa Clara, CA, USA). Libraries were sequenced with the Illumina MiSeq platform (Illumina, Inc.) using v3 reagent kits. A total of 2,889,385 300-bp paired-end reads were generated.

Sequencing adapter and low-quality bases were trimmed (bbduk), and overlapping pairs were merged (bbmerge) using the bbtools software suite (http://jgi.doe.gov/data-and-tools/bbtools/). Preprocessed reads were assembled using SPAdes v3.10.1 (5) and polished with Pilon v1.22 (6). Assembly resulted in 11 contigs (>1,000 bp), with an average of 580-fold genome coverage. Contigs were ordered with Mauve v2015-02-13 using GenBank accession no. NC_018588 as reference, the closest genome to *L. monocytogenes* ATCC 7644 from RefSeq, according to Mash v1.1.1 (7). The combined length of the draft genome was 2,964,284 bp, with a G+C content of 37.83%. Gene predictions and annotations were performed using the NCBI Prokaryotic Genome Annotation Pipeline (8), which predicted 3,033 genes, 2,967 coding sequences (CDS), 8 rRNAs, and 54 tRNAs. Two prophage sequences of 57.4 kb and 10 kb, respectively, were detected by PHASTER (9). Two of the 11 contigs were matching plasmid sequences.

### Accession number(s).

This whole-genome shotgun project has been deposited in DDBJ/ENA/GenBank under the accession no. NGZM00000000. The version described in this paper is the first version, NGZM01000000.
